# Shichangpu–Xiyangshen Herb Extract Alleviates Cognitive Dysfunction in Type 1 Diabetes Through Metabolism of Arachidonic Acid Cyclooxygenase and Lipoxygenase

**DOI:** 10.3390/molecules31091446

**Published:** 2026-04-27

**Authors:** Jialin Wang, Dongxue Wang, Yang Yang, Changyuan Jing, Xinrui Li, Yixuan Xin, Ying Wang, Hailong Xie

**Affiliations:** 1 College of Pharmacy, Heilongjiang University of Chinese Medicine, Harbin 150040, China; 13080224589@163.com (J.W.); 18347125489@163.com (C.J.); 13945034827@163.com (X.L.);; 2 College of Pharmacy, Harbin University of Commerce, Harbin 150028, China; fvfqt722@163.com (D.W.); syzxyy213@163.com (Y.Y.)

**Keywords:** diabetic cognitive impairment (DCI), Shichangpu–Xiyangshen herb pair, arachidonic acid pathway, PTGS2, ALOX-5

## Abstract

**Background**: Long-term diabetes mellitus may precipitate severe complications, including cognitive dysfunction. Existing research has shown that diabetic cognitive impairment (DCI) in rats is characterized by memory deterioration and a disordered arrangement of hippocampal cells. The Shichangpu–Xiyangshen herb pair (SX) effectively improved the pathological changes induced by DCI. However, the role of SX in regulating the physiological and behavioral responses to DCI remains unclear. **Methods**: We sought to determine the small-molecule metabolites of cerebrospinal fluid (CSF) and delineate the pathways to elucidate the potential mechanism of the effect of SX in the treatment of DCI by metabolomics strategies, focusing on key mechanisms. Behavioral assessments were conducted on DCI rats and the rats treated with SX, as well as an evaluation of neuronal morphology in the hippocampal region. Metabolomics was used to analyze biomarkers in cerebrospinal fluid at different time points during the development of DCI, to uncover the underlying core mechanisms of DCI, and to investigate the regulatory effects of SX on these core mechanisms. The mechanisms of SX on DCI were investigated using quantitative reverse transcription polymerase chain reaction, immunohistochemistry, Western blot, and ELISA. **Results**: The Morris water maze (MWM) and social interaction test results revealed that SX administration effectively counteracted cognitive impairments in rats with DCI while simultaneously diminishing pathological damage in the CA1, CA3, and DG hippocampal regions. Further analysis showed that SX restored the significantly reduced levels of IL-8, ROX, and TNF-α, and reduced Aβ plaque formation (as indicated by APP and BACE1 protein expression). Simultaneously, SX markedly ameliorated arachidonic acid metabolic disorders in DCI, including significant reductions in arachidonic acid (AA), PGE2, and LTB4 and reduced expression of COX-2 (PTGS2) and 5-LOX (ALOX-5). **Conclusions**: Our findings indicate that SX effectively counteracted cognitive impairment in rats with DCI by inhibiting AA metabolism through both cyclooxygenase and lipoxygenase pathways, thereby minimizing neuronal damage.

## 1. Introduction

Diabetes Mellitus (DM) is classified based on a patient’s dependence on exogenous insulin, as persistent hyperglycemia can lead to damage to various tissues [1]. In the 1960s, Miles first reported cognitive dysfunction in individuals with DM. Animal experiments have demonstrated that animal models of DM and Alzheimer’s disease (AD) exhibit common pathological features, including abnormal insulin signaling pathways and impaired hippocampal plasticity [2,3]. Extensive epidemiological studies have demonstrated that DM can lead to cognitive decline in patients. The incidence of mild cognitive impairment (MCI) is 1.5 times higher in DM patients compared to non-diabetic individuals, and the risk of developing dementia is 1.3 to 3.4 times greater. The primary manifestations include memory impairment, reduced cognitive function, decreased motor coordination, and emotional disturbances, with vascular dementia being the most prevalent [4]. Neuropsychological studies demonstrated that approximately 55% of individuals with cognitive impairment due to diabetes developed Alzheimer’s disease (AD) within three years, with the incidence rate reaching 100% after about 10 years [5]. However, the occurrence and therapeutic mechanisms of DCI remain incompletely elucidated. Notably, arachidonic acid metabolic dysregulation represents a potential DCI pathogenesis.

Previous research has shown that high levels of COX-2 lead to increased PGE2 synthesis, which may occur through non-enzymatic processes or via microsomal and perinuclear PGE synthase (mPGES), which is influenced by proinflammatory cytokines and glucocorticoids. In Alzheimer’s disease models, PGE2 has been shown to exacerbate Aβ- and TNFα-induced neurotoxicity. The arachidonic acid pathway is critically involved in the development of DCI [6]. Natural human Aβ delivered directly into the brain’s ventricular system suppresses LTP, which serves as a cellular correlate of learning and memory. Conversely, PGE2 counteracts the depression of postsynaptic membrane excitability typically caused by COX-2 inhibitors and reduces the influx of calcium ions associated with back-propagating dendritic action potentials and LTP in hippocampal dentate granule cells, suggesting that PGE2 plays a role in synaptic plasticity [7]. Researchers have identified 5-Lipoxygenase (5-LOX) as the enzyme that regulates the production of leukotrienes, fatty acid compounds that trigger adverse effects in the body. Moreover, these leukotrienes are known for initiating inflammatory responses associated with asthma and allergies. They also inhibit the production of tyrosine hydroxylase in the brain and contribute significantly to the development of conditions such as stroke, obesity, and type 2 diabetes. 5-LOX is predominantly expressed by inflammatory cells, including B-lymphocytes, mast cells, eosinophils, polymorphonuclear leukocytes, and monocytes. Upon activation by intracellular Ca^2+^ elevation and oxidative stress, 5-LOX relocates to the nuclear compartment where it interacts with 5-LOX-activating protein to catalyze the conversion of arachidonic acid into leukotrienes. Leukotrienes, once released, activate specific G-protein-coupled receptors and may also engage peroxisome proliferator-activated receptors to mediate their biological effects [8].

The dried root of *Panax quinquefolium* L., known as Xiyangshen in TCM, possesses a cold-natured and a slightly sweet and bitter taste, and it acts on the Heart, Lung, and Kidney Meridians, which makes it suitable for treating diseases induced by syndromes such as qi deficiency, yin deficiency, and heat-deficiency [9]. Pharmacological studies have demonstrated that Xiyangshen possesses multiple beneficial effects, including antineoplastic activity, cardiovascular protection, antioxidant properties, regulation of glucose and lipid metabolism, immunomodulation, anti-inflammatory effects, and enhancement of learning and memory [10,11,12,13,14,15,16]. As the main components of Xiyangshen, ginsenosides exert effects on blood glucose regulation and neuroprotection through the arachidonic acid pathway, indicating their potential as therapeutic candidates for DCI [17,18,19,20]. Furthermore, *Acorus tatarinowii* Schott., first documented in the Shennong’s Classic of Materia Medica and commonly known as Shichangpu, is a perennial herbaceous plant that has a bitter and spicy taste and warm nature. It is used to induce resuscitation, transform dampness, and harmonize the stomach [21]. In TCM, the rhizome of Acorus tatarinowii is extensively utilized to treat amnesia, stroke, dementia, depression, seizures, and mental disorders [21,22,23,24,25]. The volatile oil in Shichangpu, which is mainly composed of β-asarone, crosses the blood–brain barrier and is thus widely applied in the treatment of neurological disorders, owing to their potent antioxidant and anti-acetylcholinesterase activities [26,27]. Moreover, the combination of Xiyangshen and Shichangpu (SX) demonstrates therapeutic efficacy in addressing neurological disorders. Yizhi Xingnao Granule, a clinically proven prescription for AD, employs Xiyangshen and Shichangpu as monarch and assistant drugs, respectively, thereby enhancing its brain-tonifying and orifice-opening effects [28]. Furthermore, SX extract has been shown to ameliorate hippocampal protein abnormalities in DCI rats [29]. Therefore, this study aims to explore the potential synergistic mechanisms underlying the therapeutic effects of the effective fractions of Xiyangshen and Shichangpu extracts against the inflammatory status, high blood glucose levels, and cognitive impairment in DCI, based on their potential efficacy and the classical principles of TCM herbal compatibility.

## 2. Results

### 2.1. Qualitative Analysis Chemical Components in SX Extract

**Figure 1:**
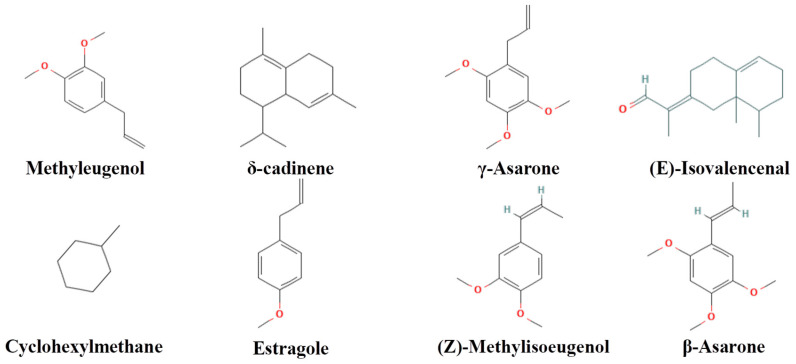
Structures of the qualitative identified compounds in Shichangpu.

**Figure 2:**
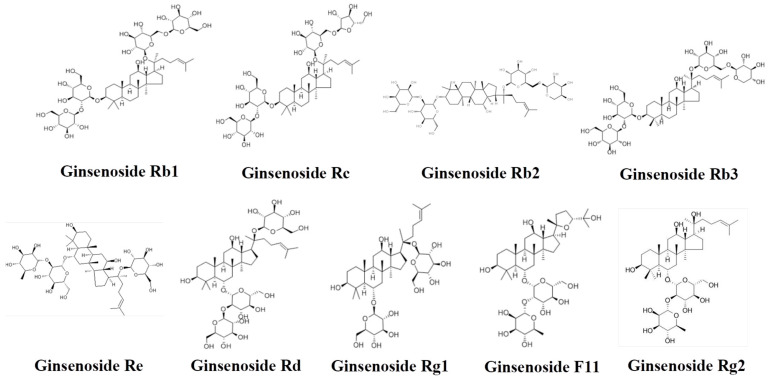
Structures of the qualitative identified compounds in Xiyangshen.

**Figure 3:**
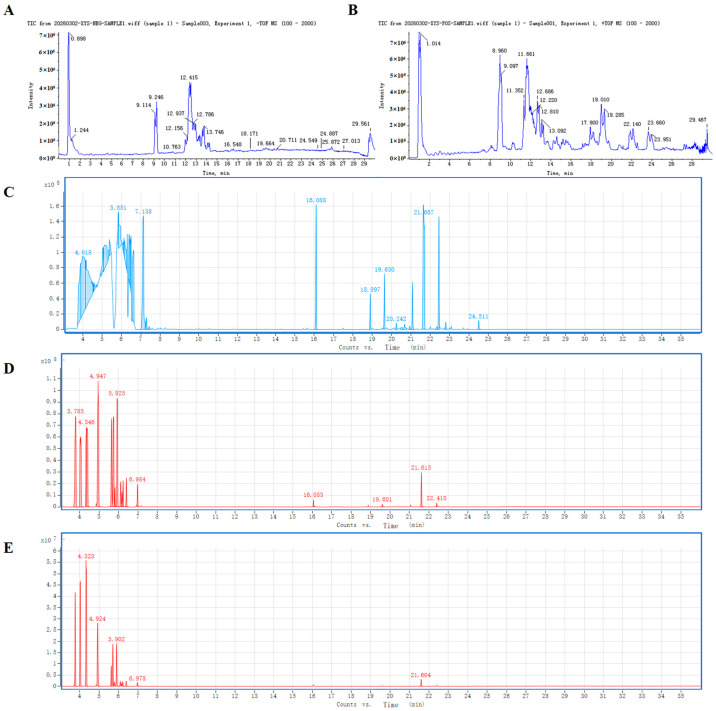
Component analysis spectrum. (**A**) TCI of UPLC−MS in negative ion mode; (**B**) TCI of UPLC−MS in positive ion mode; (**C**) TCI of HS−GCMS in splitless mode; (**D**) TCI of HS−GCMS in 20 split ratio; (**E**) TCI of HS−GCMS in 100 split ratio.

Qualitative analysis of SX extract was performed, and the chemical structures, total ion chromatograms (TICs) and compound information of nonvolatile and volatile components are presented in Figure 1, Figure 2 and Figure 3, Tables S1 and S2. A total of 17 compounds were identified, including ginsenoside Rb1 and γ-asarone.

### 2.2. Quantitative Analysis of Active Chemical Components in SX Extract

Quantitative analysis of ginsenoside Re, Rb1, Rb2, Rb3, Rg1, Rc, Rd, and pseudoginsenoside-F11 (PF11) in SX extract was performed, and their contents were determined (Table 1). Ginsenoside Rb1 and Re were the dominant ginsenosides, with concentrations of 43.90 ± 1.23 mg/g and 14.54 ± 0.87 mg/g dw, respectively.

### 2.3. SX Improved Spatial Learning and Memory Function in DCI Rats

**Figure 4:**
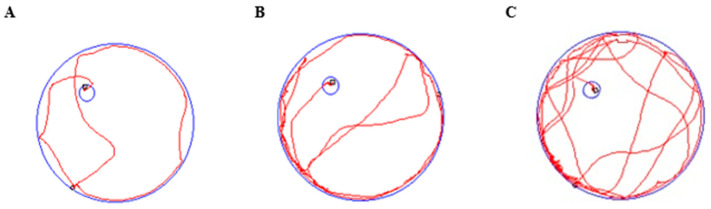
Representative swimming trajectories of rat MWM experiment: (**A**) control group; (**B**) U-DCI group; (**C**) DCI group. The blue lines represent the quadrant pool and platform area, and the red line indicates the swimming trajectory.

The Morris water maze (MWM) test was conducted to assess the cognitive impairment in DM model and the impact of SX on DCI rat. There were no significant differences in locomotor speed among the groups in the MWM test (*p* > 0.05), indicating no significant differences in physical fitness among the groups (Table 2, Figure 3C). The control group and the un-DCI model (U-DCI) group exhibited more distinct swimming trajectories, and the DCI group exhibited chaotic swimming trajectories (Figure 4). A total of 8 DCI rats were successfully modeled.

The statistical analysis comparing crossing in the platform quadrant on day 5 is illustrated in Figure 4A. Rats in the control group exhibited significantly more platform crossing times (6.0 ± 1.4 time) compared to the DCI model group (2.0 ± 1.2 time, *p* < 0.001). The positive (5.0 ± 1.3 time, *p* < 0.001), SX-Low (4.0 ± 1.5 time, *p* < 0.01), and SX-High (2.7 ± 0.3 time, *p* < 0.001) groups exhibited a significantly higher number of crossing times compared to the DCI model group. A notable difference in escape latency time was observed in the DCI group on day 5 (54.9 ± 12.7 s) compared with the control group (20.9 ± 4.1 s, *p* < 0.001), which indicated impaired cognitive function in the diabetic rats. Notably, escape latency was significantly reduced in the positive (23.5 ± 5.0, *p* < 0.001), SX-Low (29.8 ± 4.6, *p* < 0.001), and SX-High (24.7 ± 5.6, *p* < 0.001) groups compared with the model group (Figure 3B). These experimental findings indicate that SX treatment ameliorated cognitive function decline in DCI rats.

### 2.4. SX Improved Social Behavioral Deficits in DCI Rats

DCI model rats had impaired sociability in the three-chamber sociability test. Rats in the DCI model group exhibited significantly reduced contact time and frequency (contact time: 92.8 ± 21.3 s, frequency of contact: 7.6 ± 2.4) compared to the control group (contact time: 136.1 ± 12.5 s, *p* < 0.001, frequency of contact: 16.5 ± 2.9, *p* < 0.001). The positive (contact time: 138.7 ± 14.2 s, *p* < 0.001, frequency of contact: 16.4 ± 3.1, *p* < 0.001), SX-Low (contact time: 121.6 ± 14.5 s, *p* < 0.005, frequency of contact: 12.3 ± 3.4, *p* < 0.005), and SX-High (contact time: 132.1 ± 13.1 s, *p* < 0.001, frequency of contact: 15.8 ± 3.1, *p* < 0.001) groups exhibited a significantly higher number of contact time and frequency compared to the DCI model group (Figure 3D,E). These data indicate that SX improved social deficits in DCI rats.

### 2.5. SX Improved Blood Glucose Levels in DCI Rats

**Figure 5:**
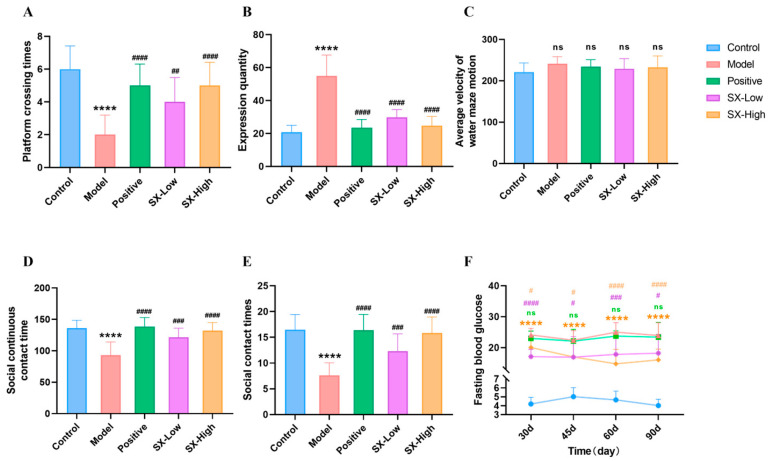
Behavioral experiment and blood glucose: (**A**) platform crossing times of MWM; (**B**) escape latency of MWM; (**C**) average velocity of MWM; (**D**) social continuous contact time; (**E**) social contact times; (**F**) fasting blood glucose. All statistical data are presented as mean ± SD, ANOVA analysis. Compared with control group: ns *p* > 0.05, **** *p* < 0.0001; compared with DCI group: ns *p* > 0.05, # *p* < 0.05, ## *p* < 0.01, ### *p* < 0.005, #### *p* < 0.001, analyzed by Student’s *t*-test.

One week following STZ injection, defined as the first day of DM model formation, the model group demonstrated significantly elevated blood glucose levels at 1 day (25.86 ± 4.39, *p* < 0.01), day 30 (24.10 ± 2.98, *p* < 0.01), 45 day (22.41 ± 3.12, *p* < 0.01), day 60 (25.01 ± 3.07, *p* < 0.01), and day 90 (23.94 ± 4.19, *p* < 0.01) compared with the control group (day 1: 7.39 ± 3.10, day 30: 4.19 ± 1.02, day 45: 5.01 ± 1.02, day 60: 4.65 ± 0.97, day 90: 4.02 ± 0.87) (Figure 5F). The positive group and SX-Low rats did not significantly reduce blood glucose levels. Treatment with SX-High significantly reduced the blood glucose in DCI rats at day 60 (14.76 ± 7.23, *p* < 0.5) and day 90 (16.03 ± 3.99, *p* < 0.5) compared with the model group (Figure 5F).

### 2.6. SX Improved Biochemical Parameters, Key Proteins in the Aβ Pathway and Histopathology in DCI Rats

**Figure 6:**
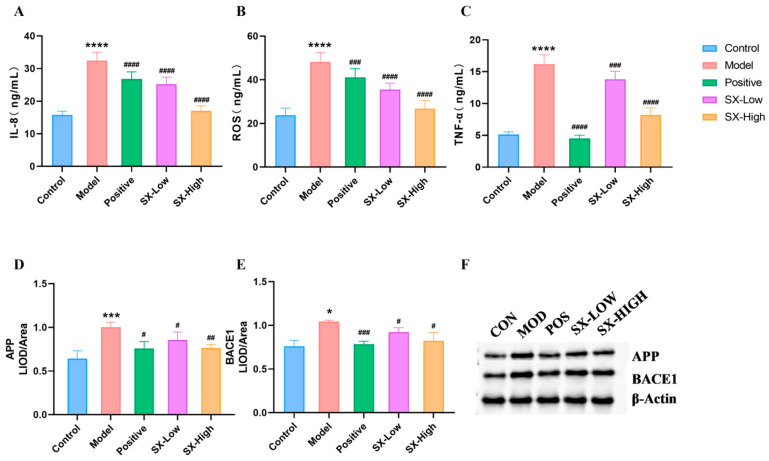
The biochemical parameters and proteins used to evaluate the efficacy of SX in the treatment of DCI. Error bars represent the mean ± SD: (**A**) IL-8; (**B**) ROS; (**C**) TNF-α; (**D**) APP mRNA expression; (**E**) BACE1 mRNA expression; (**F**) Western blot band of APP and BACE1. All statistical data are presented as mean ± SD, ANOVA analysis. Compared with control group: * *p* < 0.05, ***p < 0.005, **** *p* < 0.0001; compared with DCI group: # *p* < 0.05, ## *p* < 0.01, ### *p* < 0.005, #### *p* < 0.001; analyzed by Student’s *t*-test.

Compared with the control group (IL-8: 15.8 ± 1.2, ROS: 23.8 ± 3.2, TNF-α: 5.1 ± 0.4), the levels of IL-8 (32.5 ± 2.6, *p* < 0.001), ROS (458. ± 4.3, *p* < 0.001) and TNF-α (16.2 ± 1.5, *p* < 0.001) in the model group increased significantly. However, the trends of the above indexes were reversed after donepezil (IL-8: 26.8 ± 2.2, *p* < 0.001, ROS: 41.0 ± 4.1, *p* < 0.005, TNF-α: 4.5 ± 0.5, *p* < 0.001), SX-Low (IL-8: 25.2 ± 2.2, *p* < 0.001, ROS: 35.5 ± 3, *p* < 0.001, TNF-α: 13.8 ± 1.3, *p* < 0.005) and SX-High (IL-8: 17.0 ± 1.6, *p* < 0.001, ROS: 26.8 ± 3.7, *p* < 0.001, TNF-α: 8.2 ± 1.1, *p* < 0.001) treatment. Moreover, we found that the protein levels of APP (1.00 ± 0.05, *p* < 0.005) and BACE1 (1.04 ± 0.02 *p* < 0.05) in the hippocampus of DCI rats were increased significantly, and donepezil (APP: 0.76 ± 0.08, *p* < 0.05, BACE1: 0.78 ± 0.03, *p* < 0.001), SX-Low (APP: 0.86 ± 0.09, *p* < 0.05, BACE1: 0.92 ± 0.05, *p* < 0.05) and SX-High (APP: 0.76 ± 0.04, *p* < 0.01, BACE1: 0.82 ± 0.10, *p* < 0.05) significantly reduced the elevation of APP and BACE1 in DCI rats (Figure 6).

### 2.7. SX Improved Histopathology of Hippocampus in DCI Rats

**Figure 7:**
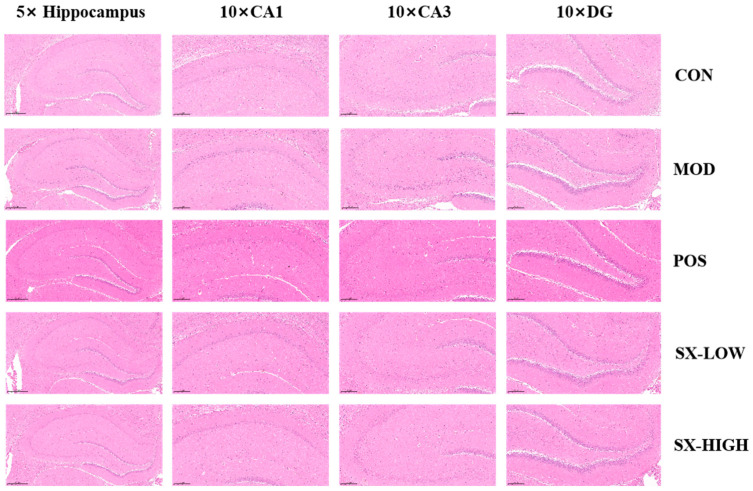
Histopathological analysis of hippocampus. 5×: Scale bar = 500 μm; 10×: Scale bar = 200 μm.

The Cornu Ammonis (CA) 1, CA3, dentate gyrus (DG) regions of the hippocampus and the cerebral cortex were subjected to pathological assessment through hematoxylin and eosin (H&E) staining. As depicted in Figure 7, the hippocampal neurons of rats in the control group exhibited clear and evenly distributed morphology, characterized by a large and distinct nucleus. The model group exhibited significant histological damage, with extensive neuronal loss, disorganized cellular architecture, pyknotic nuclei and hyperchromatic neurons in the CA1 and CA3 regions, a notable decrease in the granular layer density, pronounced interstitial thinning and glial cell proliferation in the DG. The SX-Low group showed a certain degree of cell density recovery in CA1, CA3, and DG, but the overall damage remained relatively significant. Donepezil and SX-High significantly improved the histomorphology of the hippocampus of DCI rats, and neurons in the hippocampus exhibited a well-organized arrangement, mild focal damage and residual hypertrophic cells.

### 2.8. Altered Metabolites at Multiple Time Points in DCI

**Figure 8:**
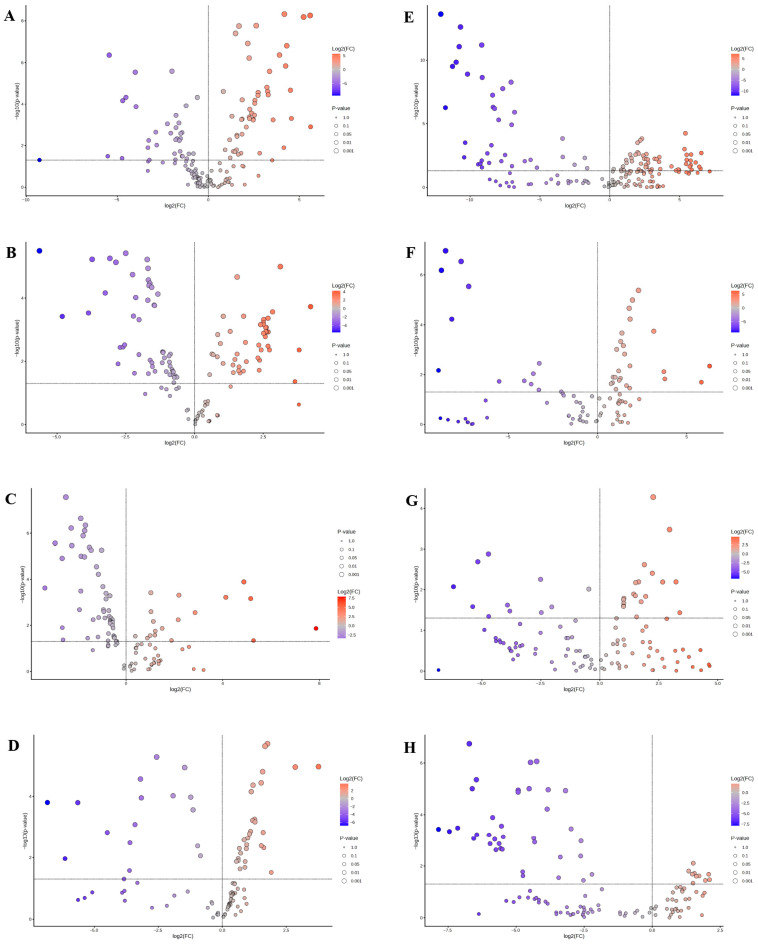
Volcano maps of DCI at days 30, 45, 60, and 90 after formation of DM. (**A**). Volcano plot in negative ion mode on day 30; (**B**). Volcano plot in positive ion mode on day 30; (**C**). Volcano plot in negative ion mode on day 45; (**D**). Volcano plot in positive ion mode on day 45; (**E**). Volcano plot in negative ion mode on day 60; (**F**). Volcano plot in positive ion mode on day 60; (**G**). Volcano plot in negative ion mode on day 90; (**H**). Volcano plot in positive ion mode on day 90; The abscissa log2FC shows the fold change value for the differences in metabolite expression between the two groups, and the ordinate log10 (*p* value) shows the results from the statistical analysis of the differences in metabolite expression. A higher value indicates a more significant difference in expression. Each point in the figure represents a specific metabolite. The size of the point represents the variable importance in the projection (VIP) value. The points on the left and right sides and the upper side represent significant differences.

**Figure 9:**
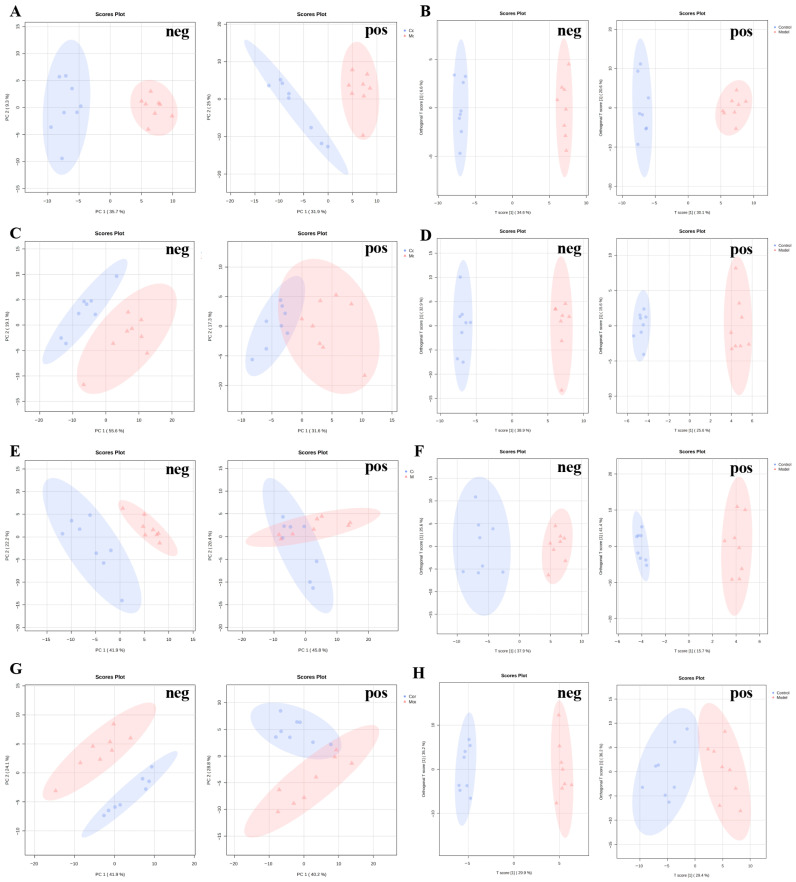
PCA and OPLS−DA spots for DCI and control group. (**A**). PCA plot for day 30 after formation of DM in the negative and positive ion modes, R2X of neg = 0.760 R2X of pos = 0.510; (**B**). OPLS−DA plot for day 30 after formation of DM in the negative and positive ion modes, Q2 of neg = 0.931 Q2 of pos = 0.897; (**C**). PCA plot for day 45 after formation of DM in the negative and positive ion modes, R2X of neg = 0.510 R2X of pos = 0.278; (**D**). OPLS−DA plot for day 45 after formation of DM in the negative and positive ion modes, Q2 of neg = 0.712 Q2 of pos = 0.800; (**E**). PCA plot for day 60 after formation of DM in the negative and positive ion modes, R2X of neg = 0.556 R2X of pos = 0.307; (**F**). OPLS−DA plot for day 60 after formation of DM in the negative and positive ion modes, Q2 of neg = 0.821 Q2 of pos = 0.415; (**G**). PCA plot for day 90 after formation of DM in the negative and positive ion modes, R2X of neg = 0.445 R2X of pos = 0.536; (**H**). OPLS−DA plot for day 45 after formation of DM in the negative and positive ion modes, Q2 of neg = 0.860 Q2 of pos = 0.703.

UPLC-MS/MS analysis revealed a grand total of 2842 negative ion peaks and 2750 positive ion peaks. Following rigorous quality control measures—including retention time alignment, elimination of missing values, and filtering out features with a fold change greater than 1 and a *p* value below 0.05 (as illustrated in Figure 8)—we identified 375 distinct metabolites. Quality control samples exhibited robust clustering in our principal component analysis (PCA) (Supplementary Figure S1), indicating high stability of the UPLC–MS/MS platform. We evaluated several multivariate analytical methods, including PCA and orthogonal PLS-DA (OPLS-DA). The quality control metrics are detailed in Supplementary Table S3. The R^2^X and Q^2^ parameters indicate good model fit. As a result, our metabolomics research is deemed to have outstanding interpretability and predictive power. The PCA and OPLS-DA visualizations depicted in Figure 9 reveal distinct clustering between the control and DCI cohorts, clearly delineating the two groups.

**Figure 10:**
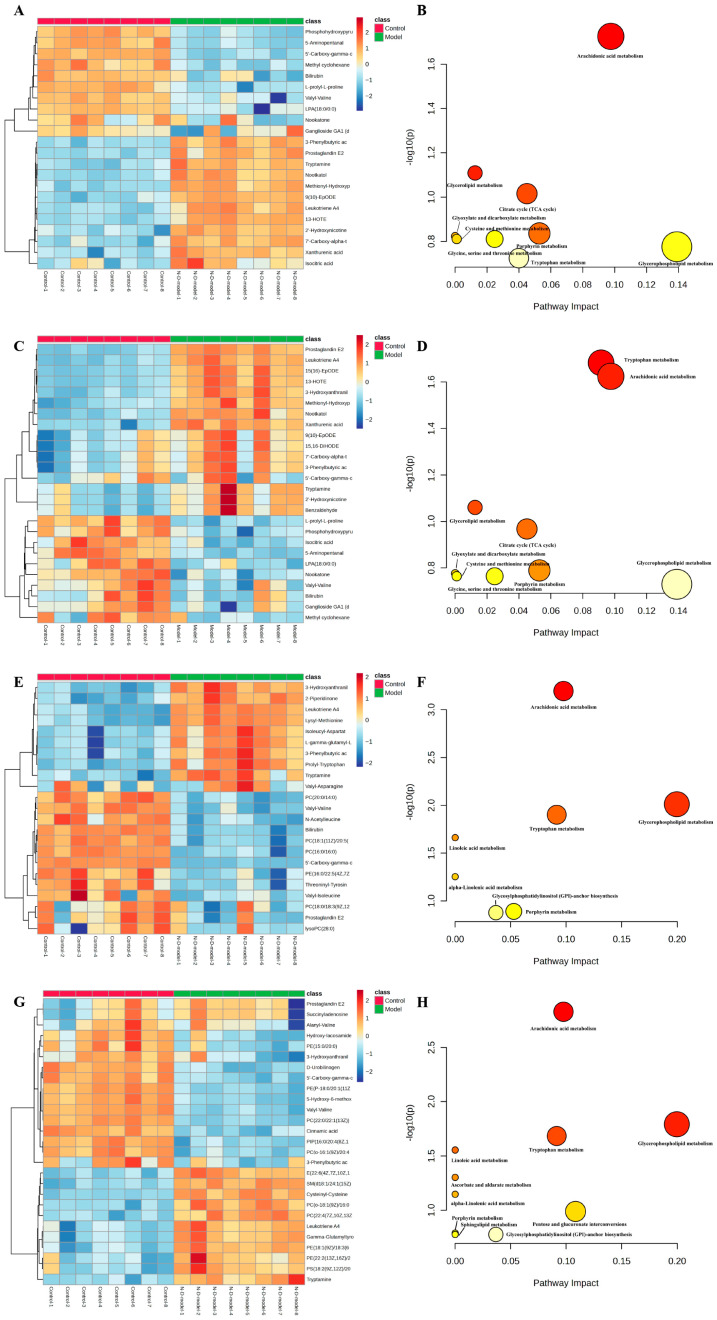
Metabolite heat map and metabolic pathway bubble diagram: (**A**,**B**) 30 days after formation of DM; (**C**,**D**) 45 days after formation of DM; (**E**,**F**) 60 days after formation of DM; (**G**,**H**) 90 days after formation of DM.

Consequently, our analysis revealed significant fluctuations in 22, 26, 23, and 27 core metabolites at the 30-, 45-, 60-, and 90-day marks following the onset of DB, respectively. Detailed information on these metabolites is provided in Supplementary Tables S4, S6, S8, and S10. To provide a clear visual representation of the varying metabolite levels throughout different stages of DCI, we generated heatmaps (Figure 10A,C,E,G).

Metabolites were analyzed through MetaboAnalyst 6.0 and cross-referenced with KEGG pathway data. This resulted in 9, 9, 7, and 10 metabolic pathways being enriched at 30 days, 45 days, 60 days, and 90 days following DM induction, respectively. Supplementary Tables S5, S7, S9, and S11 contain a comprehensive overview of all significantly enriched pathways, which are visually depicted through metabolic pathway bubble diagrams, as illustrated in Figure 10B,D,F,H. The arachidonic acid pathway was enriched at multiple time points during DCI, indicating its critical role in the pathogenesis of DCI.

### 2.9. SX Modulated Metabolites and Metabolic Contour in DCI Rats

**Figure 11:**
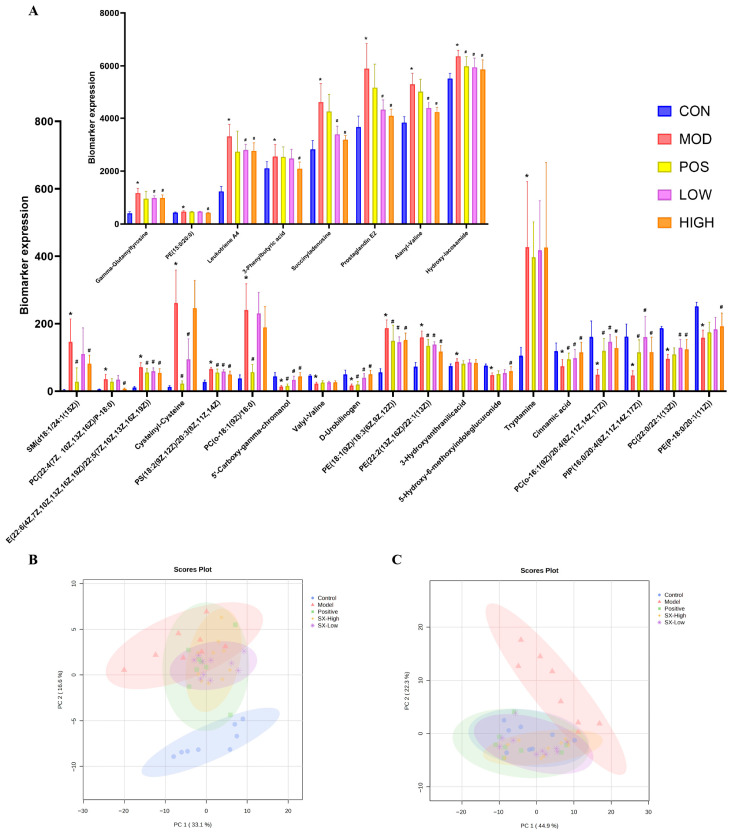
Changes in the DCI biomarkers levels and metabolic contours. Error bars represent the mean ± SD: (**A**) DCI biomarkers levels; (**B**) PCA plot in negative ion mode; (**C**) PCA plot in positive ion mode. * *p* < 0.05; compared with DCI group: # *p* < 0.05; analyzed by Student’s *t*-test.

As shown in the PCA score plot (Figure 11B,C), compared with the DCI group, the donepezil, SX-Low, and SX-High groups restored metabolic profiles closer to those of the control group. In addition, as shown in the DCI biomarkers levels, donepezil, SX-Low, and SX-High significantly adjusted 10, 17, and 22 DCI biomarkers, suggesting that high-dose SX exhibits superior efficacy in DCI.

### 2.10. SX Modulated Arachidonic Acid Pathway in DCI Rats

**Figure 12:**
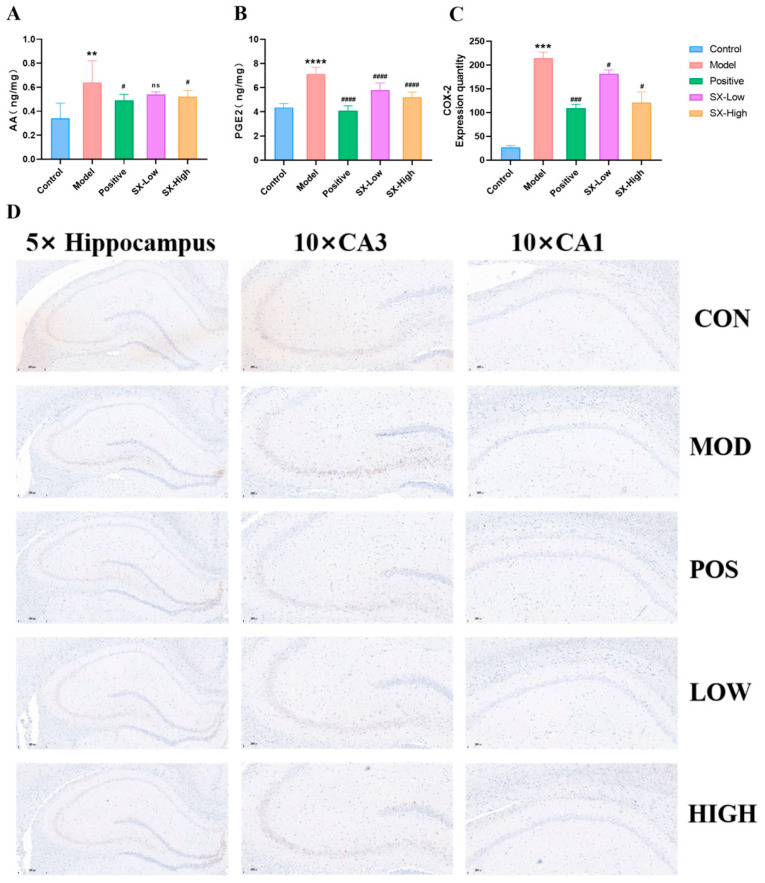
SX restores arachidonic acid cyclooxygenase pathway in DCI rats. (**A**) ELISA testing of arachidonic acid (AA); (**B**) ELISA testing of prostaglandin E2 (PGE2); (**C**,**D**) immunohistochemical testing for COX-2 (n = 3). All statistical data are presented as mean ± SD, ANOVA analysis. Compared with control group: ** *p* < 0.01, *** *p* < 0.005, **** *p* < 0.0001; compared with DCI group: # *p* < 0.05, ### *p* < 0.005, #### *p* < 0.001; analyzed by Student’s *t*-test. 5×: Scale bar = 500 μm; 10×: Scale bar = 200 μm.

**Figure 13:**
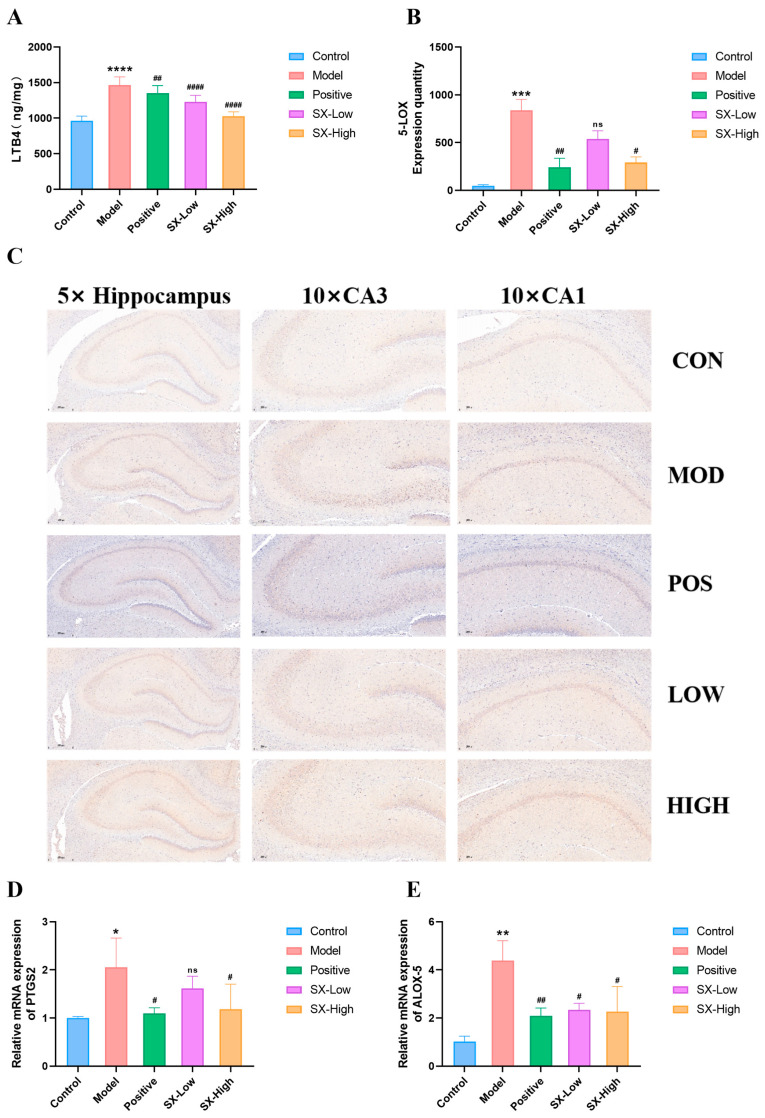
SX restores arachidonic acid lipoxygenase pathway in DCI rats. (**A**) ELISA testing of leukotriene B4 (LTB4); (**B**,**C**) immunohistochemical testing for 5-LOX (n = 3); (**D**,**E**) qRT-PCR testing of ALOX-5 and PTGS2 (n = 3). All statistical data are presented as mean ± SD, ANOVA analysis. Compared with control group: * *p* < 0.05, ** *p* < 0.01, *** *p* <0.005, **** *p* <0.0001; compared with DCI group: # *p* < 0.05, ## *p* < 0.01, #### *p* < 0.001; analyzed by Student’s *t*-test. 5×: Scale bar = 500 μm; 10×: Scale bar = 200 μm.

ELISA testing revealed that DCI rats markedly upregulated the levels of AA (0.34 ± 0.13, *p* < 0.005), PGE2 (7.1 ± 0.5, *p* < 0.001), and LTB4 (1465.1 ± 116.4, *p* < 0.001), while donepezil (AA: 0.49 ± 0.05, *p* < 0.05, PGE2: 4.1 ± 0.4, *p* < 0.001, LTB4: 1350.2 ± 76.0, *p* < 0.01), SX-Low (AA: 0.54 ± 0.10, *p* > 0.05, PGE2: 5.8 ± 0.6, *p* < 0.001, LTB4: 1230.8 ± 89.9, *p* < 0.001), and SX-High (AA: 0.52 ± 0.05, *p* < 0.05, PGE2: 5.2 ± 0.4, *p* < 0.001, LTB4: 1025.6 ± 62.8, *p* < 0.05) remarkably suppressed the upregulation of AA, PGE2, and LTB4 levels (Figure 12A,B and Figure 13A). Moreover, DCI rats significantly increased the COX-2 (214.0 ± 12.7, *p* < 0.001) and 5-LOX (838.0 ± 115.4, *p* < 0.005) proteins expressed in DCI rats, whereas donepezil (COX-2: 109.0 ± 8.6, *p* < 0.005, 5-LOX: 241.9 ± 94.5, *p* < 0.01) and SX-High (COX-2: 120.8 ± 22.0, *p* < 0.05, 5-LOX: 291.6 ± 58.9, *p* < 0.05) treatment reduced protein levels of COX-2 and 5-LOX in DCI rats, and SX-Low treatment selectively reduced protein levels of COX-2 in DCI rats (COX-2: 181.6 ± 8.2, *p* < 0.05, 5-LOX: 539.2 ± 84.3, *p* > 0.05, Figure 12C,D and Figure 13B,C). Importantly, DCI rats showed significantly increased mRNA expression of PTGS2 (2.1 ± 0.6, *p* < 0.05) and ALOX-5 (4.4 ± 0.8, *p* < 0.01), whereas donepezil (PTGS2: 1.1 ± 0.1, *p* < 0.05, ALOX-5: 2.1 ± 0.3, *p* < 0.01), and SX-High (PTGS2: 1.0 ± 0.3, *p* < 0.05, ALOX-5: 2.3 ± 1.0, *p* < 0.5) treatment reduced mRNA levels of ALOX-5 and PTGS2 in DCI rats, and SX-Low treatment selectively reduced protein level of ALOX5 in DCI rats (PTGS2: 1.7 ± 0.1, *p* > 0.05, ALOX-5: 2.3 ± 0.3, *p* < 0.05, Figure 12C,D and Figure 13B–E). Overall, the data implies that arachidonic acid signaling pathway is activated in DCI rats, contributing to cognitive function in diabetic rats.

## 3. Discussion

Several studies have indicated the beneficial influence of SX on cognitive impairment in diabetic rats, and arachidonic acid metabolism is usually enriched in the occurrence of DCI [29,30]. However, the role of SX in alleviating glucose and cognitive levels in DCI remains unclear. Here, we show that SX treatment significantly ameliorated hyperglycemia, learning, memory, and social behavior in the DCI model, clarifying the effect of SX on DM and cognitive impairment in the DCI model. According to biochemical indicators, SX reduced the levels of IL-8, ROS, TNF-α, APP, and BACE1 in DCI rats. These findings suggest that this herbal pair can regulate inflammatory factor disorders and the accumulation of Aβ plaques through the pathological changes of tissues. It was also found that SX has a protective effect on the CA1, CA2, and DC regions of the hippocampus, attenuating hippocampal pathological damage. Employing multi-timepoint non-targeted metabolomics analysis, we observed a significant enrichment of abnormal metabolism of arachidonic acid at 30, 45, 60, and 90 days after DM modeling in the DCI model group, indicating the significant effect of arachidonic acid metabolism in the development process of DCI. Subsequently, we identified 27 core differential metabolites and 10 specific metabolic pathways that were differentially expressed between the control and DCI groups, and SX significantly reversed the core metabolites in arachidonic acid metabolism, implicating this pathway as a key therapeutic target. Finally, the results of ELISA, IHC, and qRT-PCR suggest that SX significantly adjusted AA, PGE2, and LTB4 in serum, AA metabolic enzymes in the hippocampus (COX-2 and 5-LOX), and the mRNA level, regulating the synthesis of COX-2 and 5-LOX (PTGS2 and ALOX-5).

DCI in the early stage is primarily characterized by Yin deficiency and heat flourishing syndrome, and the moderate and late stages are mainly characterized by Qi-Yin deficiency or Yin-Yang deficiency syndrome. *Acorus tatarinowii* and *Panax quinquefolius* L. form an herbal pair with Qi-Invigorating, Kaiqiao, and Yizhi effects. The pharmacological properties of Xiyangshen, a traditional Chinese medicinal herb, particularly its anti-inflammatory, antioxidant, and neuroprotective effects, as well as its influence on insulin metabolic disorders and the nervous system, have been demonstrated in its root and extracts [13,31,32]. Similarly, Shichangpu (*Acorus tatarinowii Aiton*) exhibits comparable therapeutic properties [33]. Furthermore, Xiyangshen and Shichangpu exhibit favorable effects in preventing diabetic complications such as diabetic nephropathy, diabetic retinopathy, and hypertension [34,35,36,37]. Regarding cognitive enhancement, experimental studies have demonstrated that Xiyangshen attenuates chronic unpredictable stress-induced cognitive impairment through modulation of the nitrergic signaling pathway [38]. Learning and memory deficits induced in mice by scopolamine, morphine and methamphetamine are antagonized by Pseudoginsenoside-F11 (PF11), which is a component extracted from Xiyangshen [39]. In neurodegenerative and neurovascular diseases, importance has been attributed to β-Asarone, a major component extracted from Shichangpu. Autophagy mitigation and damage reduction in hypoxic cells, along with improvements in learning and memory function, have been confirmed as its effects [40]. With respect to blood glucose improvement, Xiyangshen treatment was associated with significant elevations in glycogen and high-density lipoprotein (HDL), as well as notable decreases in both plasma cholesterol and low-density lipoprotein (LDL) concentrations [41]. Furthermore, it has an anti-diabetic potential as it increases insulin sensitivity [42]. Isolated from the rhizomes of Shichangpu are tatanans, which belong to complex sesquilignan natural products. Potent glucokinase-activating properties of tatanans A, B and C have been previously reported, with in vitro activity surpassing that of known synthetic antidiabetic agents [43]. It is evident that these findings collectively substantiate the protective effects of Xiyangshen and Shichangpu against hyperglycemia and cognitive impairment; however, further investigation is required to elucidate the mechanism by which the drug pair of Shichangpu and Xiyangshen operates in cognitive impairment induced by diabetes. In this study, we assert that the SX herbal pair exhibits neuroprotective and antidiabetics activities in the DCI model through improved histopathology of the hippocampus, reducing abnormally elevated levels of inflammatory cytokines and Aβ plaque formation, and modulating metabolism of arachidonic acid via lipoxygenase and cyclooxygenase pathways in DCI rats.

Evidence suggests that hippocampal neurons could be injured by high glucose exposure and that this neuronal injury is potentially mediated through intracellular ROS accumulation [44]. Clinical data have demonstrated the atrophy of the right CA1, low volume of the left DG, and microgliosis and astrogliosis in the CA1, CA2, and CA3 regions of hippocampus [44,45,46,47]. In addition, elevated proinflammatory cytokines TNF-α and IL-1β in the hippocampus or serum are related to memory impairment [48]. Compared to the model group, rats in the SX-High and SX-Low groups exhibited improved morphological characteristics of the hippocampus, with regular arrangement of neurons and fewer constrictor cells, highlighting the effective therapeutic functions of SX in improving neuronal damage. SX also improved the inflammatory cytokine levels associated with cognitive impairment in the DCI model. Therefore, SX may improve cognitive impairment in DCI and potentially regulate hippocampal structure and inflammatory factor levels.

Experimental studies have demonstrated that the arachidonic acid metabolism pathway may play significant roles in cognitive disorder [49,50]. PGE2, which is derived from AA via COX-2 within the AA metabolic pathway and functions via EP receptors, promotes neurogenesis in adult neurogenic niches of the telencephalon. Manipulation of this signaling pathway has been suggested as a potential means by which neurogenesis might be promoted under pathological conditions [51]. 5-LOX, a proinflammatory enzyme, serves as an important modulator in the development of age-related neuropathology, neurocyte inflammation, and apoptosis. Increased within the brains of aged rats and reaching high levels in cognitively impaired animals, this enzyme demonstrates age-dependent expression patterns [52,53,54]. PTGS2 and ALOX-5 encode the expression of COX-2 and LOX5, respectively [55,56]. As a result, regulation of COX-2 (PTGS2) and 5-LOX (ALOX-5), which are critical regulators of neuroinflammation, might represent a crucial therapeutic target for DCI intervention. Therefore, the mediating role of arachidonic acid metabolism—specifically its regulation through cyclooxygenase and lipoxygenase pathways—in the cognitive impairment induced by DCI constituted the focus of the present study. Our results indicate that treatment with SX can lead to the downregulation of AA, PGE2, and LTB4, suggesting that SX can be a potent inhibitor of arachidonic acid metabolism. Furthermore, SX consumption further reduced COX-2 (PTGS2) and 5-LOX (ALOX-5) expression, supporting the hypothesis that SX suppresses cognitive disorders of DCI through multi-target modulation of AA metabolism.

Admittedly, this study also suggests that SX may improve DCI through multiple mechanisms, including blood glucose regulation, anti-inflammatory effects, oxidative stress inhibition, and metabolic disorder correction; the underlying mechanism remains to be further elucidated. However, it is essential to interpret these pleiotropic effects within the context of the specific animal model employed. Although the STZ-induced diabetic cognitive impairment model used in this study effectively replicates core pathological features observed clinically (hyperglycemia, hippocampal neuronal damage, and cognitive decline), it is based on type 1 diabetes mellitus. Future studies should employ animal models more closely resembling type 2 diabetes mellitus (e.g., high-fat diet combined with low-dose STZ) to comprehensively elucidate the mechanisms of SX in DCI treatment and enhance its clinical translational value.

## 4. Materials and Methods

### 4.1. Drug Preparation

#### 4.1.1. Citric Acid–Sodium Citrate Solution

First, 2.10 g citric acid was dissolved and diluted with distilled water to 100 mL as a citric acid (A) solution. Similarly, 2.94 g sodium citrate was dissolved and diluted with distilled water to 100 mL as a sodium citrate (B) solution. Solutions A and B were uniformly mixed at a ratio of 1:1.32, with the pH adjusted to a range of 4.2–4.5. The mixture was then passed through a microporous filter membrane (Φ = 0.22 μm) for sterilization and stored at 4 °C until use.

#### 4.1.2. Streptozotocin (STZ) Solution

The STZ powder was dissolved in a citric acid–sodium citrate buffer solution to prepare a 1% STZ solution, which was stored in a cool, light-protected place, and administered within 10 min after preparation.

#### 4.1.3. Extract of Shichangpu–Xiyangshen (SX)

First, 100 g of Shichangpu powder (filtered through a 40-mesh sieve) was accurately weighed and soaked in 800 mL of distilled water for 2 h. It was subsequently reflux-extracted for 9 h using a volatile oil extractor, with the collected volatile oil serving as the active component of Shichangpu. Then, 150 g of Xiyangshen powder (filtered through a 40-mesh sieve) was accurately weighed and refluxed twice with 1800 mL of 70% ethanol for 3 h each. The filtrate was concentrated at 60 °C to approximately 750 mL, from which the aqueous solution was lyophilized to obtain the powder. All extracts of Shichangpu and Xiyangshen were uniformly mixed, and 5 g of the mixture was accurately weighed and dissolved in 50 mL of water to prepare a 0.1 g/mL Shichangpu–Xiyangshen (SX) extract, which was stored at 4 °C and subjected to ultrasonic treatment prior to intragastric administration.

### 4.2. Component Analysis of SX Extract

#### 4.2.1. Analysis of Non-Volatile Components

An AB SCIEX Triple TOF™ 5600 UPLC-MS (Allen Bradley, Milwaukee, WI, USA) system was used for qualitative analysis. Chromatographic separation was achieved on a Waters C18 analytical column (2.1 mm × 100 mm, 1.7 μm, Waters, Milford, MA, USA) using a gradient elution system with a mobile phase of 0.1% aqueous formic acid (A) and 0.1% formic acid in acetonitrile (B) at a flow rate of 0.3 mL/min. The gradient elution program was as follows: 10% B at 0 min, 100% B at 27 min, 10% B at 30 min. The column temperature was 30 °C and the injection volume was 2 μL.

The MS conditions were as follows: data were collected in the positive and negative ion modes from 100 to 1200 *m*/*z*; ion source temperature: 120 °C, desolvation temperature: 300 °C; capillary voltage: 2.5 kV (ESI^+^) and 2.0 kV (ESI^−^); declustering potential voltage: 40 V; desolvation gas flow rate: 800 L/h.

Data acquisition was performed using Analyst TF 1.7.1 software, and data processing was conducted with PeakView 1.2 software. MS data were preferentially matched against the Natural Products HR-MS/MS Spectral Library 1.0. Compounds were initially screened based on the score information of each chromatographic peak and subsequently confirmed by MS and MS/MS data of corresponding peaks. Compounds not included in the database were identified by reference to the published literature and MS fragmentation patterns.

#### 4.2.2. Analysis of Volatile Components

Volatile oil components of SX extract were analyzed by HS-GCMS (AHS-7900E, Beijing Zhongyi Yusheng Technology Co., Ltd., Beijing, China), equipped with a J & W DB-5MSD capillary column (15 mm × 0.20 mm, 0.25 μm film thickness). The temperature of the injector was 270 °C, and helium was the carrier gas at 7 psi. The identification of the components was compared with the spectral library. Compounds with match scores ≥80% were considered identified.

### 4.3. UHPLC-HRMS Quantitative Analysis of Active Chemical Components in SX Extract

Ginsenosides were identified as the main active components of the SX extract, including ginsenoside Re (batch number: MUST-14032301), Rb1 (batch number: MUST-14032301), Rb2 (batch number: MUST-14072210), Rb3 (batch number: MUST-14072211), Rg1 (batch number: MUST-15042215), Rc (batch number: MUST-14091710), Rd (batch number: MUST-15020910), and PF11 (batch number: MUST-14041212) (manufacturer: Chengdu Must Bio-Technology Co., Ltd., Chengdu, China). These were quantitatively analyzed using UPLC-HRMS (UPLC-I-CLASS Xevo TQD, Waters, USA) for quality control and characterization of their ginsenoside profiles. Then they were analyzed by a C18 chromatographic column (Waters ACQUITY BEH C18, 2.1 mm × 100 mm, 1.7 μm), with 0.1% formic acid in water as mobile phase A and acetonitrile as mobile phase B. Gradient elution was conducted at a flow rate of 250 µL/min and a column temperature of 40 °C. The gradient changes in mobile phase A were as follows: 0–0.5 min (25–25%), 0.5–2 min (25–50%), 2–5 min (50–60%), 5–5.5 min (60–25%), 5.5–7 min (25–25%).

Ginsenosides were analyzed using the multiple reaction monitoring (MRM) mode. The mass spectrometry analysis conditions were as follows: ion source temperature, 350 °C; capillary voltage, 2.8 kV; desolvation gas temperature, 650 °C; desolvation gas flow, 500 L/h; cone gas flow, 50 L/h; and nebulizer pressure, 7.0 bar.

### 4.4. Animals

Changchunshi Yisi Experimental Animals Technology Co., Ltd. (Changchun, China, certificate number: 20158165) supplied specific pathogen-free (SPF) male Wistar rats, aged 9–11 months and weighing 250 ± 20 g. For seven consecutive days, these rats were given free access to food and water while being housed in a sterile room with a temperature range of 19–25 °C and a relative humidity range of 45–65%. After acclimatization, the rats were randomly assigned to the control (n = 8) and model (n = 92) groups. The sample sizes used for subsequent experiments were as follows: control, model, and positive groups, n = 8 each; SX-Low and SX-High groups, n = 10 each. The entire animal experiment was conducted using randomized, double-blind methods to ensure experimental validity.

To induce diabetes, rats in the model group received an intraperitoneal injection of streptozotocin (STZ, 50 mg/kg), while the control group rats received an equivalent volume of citric acid–sodium citrate buffer solution. After a 72 h period, tail vein blood was collected for the purpose of examining blood glucose levels. Fasting blood glucose >16.7 mmol/L was considered indicative of successful modeling in the rat, with this day being documented as the initial day of successful modeling [23]. Of the 92 rats that successfully developed diabetes, the diabetic model rats were randomly allocated into four distinct groups: the diabetes cognitive impairment group (model group, n = 25), the donepezil group (positive group, administered 3 mg/kg donepezil via gavage, n = 15), the Shichangpu–Xiyangshen (SX) low-dose group (SX-Low group, administered 0.54 g/kg SX extract via gavage, n = 15), and the SX high-dose group (SX-High group, administered 1.08 g/kg SX extract via gavage, n = 15). The treatment regimen was maintained continuously for a duration of 90 days. Throughout the experiment, a total of 25 rats perished: 0 from the control group, 8 from the model group, 7 from the positive group, 5 from the SX-Low group, and 5 from the SX-High group.

### 4.5. Morris Water Maze Test (MWM)

MWM was performed on day 90 of the experiment. All rats were placed in the water maze for 90 s to acclimate to the environment on the first day before MWM. Rats were trained twice daily at 20 min intervals for 5 consecutive days. In each trial, rats were given 90 s to find the platform. Their escape latency, platform dwell time, and swimming velocity were documented. In cases where the platform was not located within a 90 s timeframe, an escape latency of 90 s was recorded. Subsequently, the platform was removed, and rats were randomly placed in the quadrant opposite the previous platform location, facing the pool wall. The number of crossings over the former platform location and the stay period in the target quadrant were recorded as parameters for learning and memory evaluation. Cognitive impairment was defined as an escape latency of 90 s for two consecutive trials and by an escape latency that did not decrease below the average latency of the control group’s third training session for two consecutive trials [24].

### 4.6. Three-Chambered Social Approach Task

The test rat was placed in an apparatus box composed of three chambers separated by a 7 cm wide passage. After allowing the test rat to freely explore the operation box for 10 min, the target rat was randomly placed in one of the chambers. The test rat was then allowed to continue exploring for another 10 min, during which the number of contact attempts and the duration of contact between the test rat and the target rat were recorded.

### 4.7. Non-Targeted Metabolomics Analysis

#### 4.7.1. Cerebrospinal Fluid Collection and Preparation

An intraperitoneal injection of 0.3% sodium pentobarbital (45 mg/kg) was administered to anesthetize all rats. After being fixed to the stereotactic brain apparatus, a scalp needle was inserted into the cisterna magna, and CSF was slowly aspirated. CSF was collected on days 30, 45, 60, and 90 after modeling, and stored in a −80 °C freezer. After centrifugating the CSF at 4 °C and 13,000 rpm for 10 min, the resulting supernatant was collected with the objective of obtaining the CSF test sample. Subsequently, a quality control (QC) sample was prepared by mixing 10 μL of samples from each group.

#### 4.7.2. LC-MS Analysis Conditions

We used the UHPLC-QTOF-MS system from Waters as the instrument platform for this study. A volume of 5 μL of sample was separated by a Waters ACQUITY UPLC BEH C18 column (2.1 mm × 100 mm, 1.7 μm) and then subjected to mass spectrometry detection. The chromatographic conditions were set as follows: solvent A consisted of 0.1% formic acid in water, and solvent B consisted of 0.1% formic acid in acetonitrile. The solvent B gradient was changed from 5% to 35% (0–2 min), 35% to 35% (2–5 min), 35% to 60% (5–11.5 min), 60% to 100% (11.5–13.5 min), and the flow rate was 0.3 mL/min. The column temperature was maintained at 35 °C.

The data were collected with an ESI source operating in either the positive or negative ion mode, and detection was conducted over a mass range of 50 to 1500 *m*/*z*. The experimental conditions were set as follows: heater temperature, 550 °C, aux gas flow rate, 55 psi, air curtain gas 35 psi, ion-spray voltage floating (ISVF), −4500 V in the negative mode and 5500 V in the positive mode; pyrolysis voltage, −80 V in the negative mode and 80 V in the positive mode; collision energy, −35 eV in the negative mode and 35 eV in the positive mode. Data acquisition was performed in Deduct Dynamic Background (DBS) mode using the Analyst TF 1.6 workstation, and the CDS mass calibration system was used for online correction. The ion scan range of *m*/*z* 50–1500, followed by MS/MS analysis targeting the eight peaks exhibiting an IDA value exceeding 100 cps.

#### 4.7.3. Data Processing and Multivariate Statistical Analysis

Progenesis QI (Waters Corporation, Milford, MA, USA) was used to perform baseline filtering and peak recognition from the obtained raw data, and a data matrix that included the retention time, mass-to-charge ratio, and peak intensity was ultimately obtained. We used the MetaboAnalyst platform (https://www.metaboanalyst.ca/, accessed on 22 April 2026) to analyze the data. We constructed statistical models using principal component analysis (PCA), partial least squares discriminant analysis (PLS-DA), and orthogonal PLS-DA (OPLS-DA) techniques. Score plots were generated to visualize sample clustering and relationships Through OPLS-DA, we successfully identified the metabolites responsible for class differentiation based on the variable importance in projection (VIP) metric. Ultimately, metabolites with VIP > 1 and *p* < 0.05 (Student’s *t*-test) were considered statistically significant. Pathway enrichment analysis was conducted using MetaboAnalyst 6.0. Significant metabolites were mapped to the Kyoto Encyclopedia of Genes and Genomes (KEGG) database to elucidate the underlying biological pathways and metabolic mechanisms.

### 4.8. ELISA

The concentrations of prostaglandin E2 (PGE2), arachidonic acid (AA), leukotriene B4 (LTB4) in brain tissue, as well as tumor necrosis factor-alpha (TNF-α), interleukin-8 (IL-8), and Reactive Oxygen Species (ROX) in serum were measured according to the ELISA manufacturer’s instructions. Standards and samples were added to a 96-well microplate, which was then incubated at 37 °C for 30 min and subsequently washed thoroughly five times. HRP-conjugated reagent was added, followed by 30 min of incubation at 37 °C, after which the plate was washed five times. Subsequently, Chromogen Solution was added, followed by incubation in the dark at 37 °C for 10 min. Finally, the absorbance of the samples was measured at 450 nm after adding the stop solution. To determine the final concentration, the sample concentrations were first calculated using a standard curve and then adjusted by applying the appropriate dilution factor.

### 4.9. HE Staining

We placed these brain tissues in 5% buffered formalin, and then embedded in paraffin. A microtome was used to prepare 5-μm-thick sections, and stained with hematoxylin and eosin (H&E). After washing with distilled water, the sections were dehydrated through a gradient alcohol series (75%, 85%, 95%, and 100%) and clear in xylene. Prior to microscopic examination, the sections were sealed with neutral resin.

### 4.10. Western Blotting

Tissues were homogenized by sonication in ice-cold RIPA lysis buffer containing protease and phosphatase inhibitors. The samples were then centrifuged at 4 °C and 13,500 rpm for 30 min, after which the supernatant was collected. The BCA working solution was mixed with the protein dilution buffer and incubated at 37 °C for 20 min. Protein concentrations were determined using a microplate reader. Equal amounts of protein were separated by sodium dodecyl sulfate–polyacrylamide gel electrophoresis (SDS–PAGE). The proteins were transferred to a nitrocellulose membrane (NC filter), which was blocked with nonfat milk for 2 h and then incubated overnight at 4 °C with mouse monoclonal antibodies against β-secretase 1 (BACE1) and amyloid precursor protein (APP, Abcam, UK). After washing the membranes three times with PBST (5 min each time), they were incubated with the corresponding HRP-conjugated secondary antibodies at room temperature for 50 min. The proteins in the blots were scanned using an Odyssey scanner, and the bands were analyzed using ImageJ software (Version 1.53b).

### 4.11. Immunohistochemistry

Following dewaxing in xylene, the paraffin-embedded brain tissue sections were subjected to hydration via a descending alcohol concentration gradient. Antigen retrieval was subsequently performed on these sections using a citric acid buffer, followed by washing with PBS. Endogenous peroxidase activity was blocked with 3% H_2_O_2_ for 10 min, followed by overnight incubation at 4 °C with primary antibodies against COX-2 and 5-LOX. This was followed by secondary antibodies for 30 min at room temperature, after which visualization was facilitated through the use of DAB. Dehydration of the sections was using an ascending series of alcohol concentrations, and imaged at ×20 magnification. Finally, optical density (OD) values were determined using AIpathwell^®^ software (Saiviewer-2.2.1).

### 4.12. Quantitative Reverse Transcription–Polymerase Chain Reaction (qRT-PCR)

RNA was isolated from rats hippocampal tissues using ER501 TRIzol reagent (PerfectStart, Beijing, China). The EasyScript™ RT reagent kit (AE311, Beijing, China) was utilized for cDNA synthesis in strict accordance with the manufacturer’s protocol. Real-time quantitative PCR was performed on the StepOne system (Thermo Fisher Scientific, Waltham, MA, USA) in accordance with the provided guidelines. The fold change in mRNA expression was determined using the 2^−ΔΔCt^ calculation method, with primer sequences detailed in Table 3.

## 5. Conclusions

In summary, this study demonstrates that SX ameliorates cognitive deficits and hyperglycemia in DCI rats. These effects correlate with inhibition of inflammatory factors, Aβ plaque formation, the COX-2/PGE2 pathway, and 5-LOX/LTB4 pathway. These findings elucidate the mechanisms underlying the neuroprotective and antidiabetic effects of SX, highlighting its potential as a therapeutic candidate for DCI.

## Figures and Tables

**Table 1:** Ginsenoside contents in SX extract.

No.	1	2	3	4	5	6	7	8
Compounds	Ginsenoside Re	Ginsenoside Rb1	Ginsenoside Rb2	Ginsenoside Rb3	Ginsenoside Rg1	Ginsenoside Rc	Ginsenoside Rd	PF11
Molecular Formula	C_48_H_82_O_18_	C_54_H_92_O_23_	C_53_H_90_O_22_	C_53_H_90_O_22_	C_42_H_72_O_14_	C_53_H_90_O_22_	C_48_H_82_O_18_	C_42_H_72_O_14_
Content (Means ± SD) [mg/g dw]	14.54 ± 0.87	43.90 ± 1.23	6.24 ± 1.80	4.34 ± 0.65	1.71 ± 0.25	2.72 ± 0.36	7.71 ± 0.50	2.03 ± 0.28

**Table 2:** Average speed of swimming in the groups of rats (X ± *s*, mm/s, *n* = 3).

Group	1	2	3	4	5	6	7	8	9	10
Con	233.12 ± 24.31	236.85 ± 22.47	224.03 ± 23.19	231.66 ± 25.04	243.27 ± 26.11	226.94 ± 22.80	229.37 ± 23.19	221.68 ± 24.06	223.55 ± 25.71	212.47 ± 21.33
U-DCI	232.44 ± 25.60	230.71 ± 24.89	228.93 ± 22.17	227.55 ± 23.64	238.02 ± 27.05	224.19 ± 24.66	228.02 ± 22.91	226.51 ± 23.22	224.73 ± 24.39	223.84 ± 23.97
DCI	248.96 ± 26.54	252.41 ± 24.33	234.58 ± 25.17	236.92 ± 27.06	239.84 ± 24.72	235.72 ± 26.91	237.88 ± 27.45	233.40 ± 25.62	236.01 ± 24.37	229.63 ± 25.10

Con: control group; U-DCI: non-cognitive impaired diabetic group; DCI: diabetic cognitive impaired group.

**Table 3:** Primers used in research.

Gene	Direction	Primers (5′–3′)
*PTGS2*	forward	ATGTTCGCATTCTTTGCCCAG
	reverse	TACACCTCTCCACCGATGAC
*ALOX5*	forward	TGCACCGTGGTTGAAGATTCTC
	reverse	ATTGAGCCATCCTTCCAGTTGC

## Data Availability

The original contributions presented in this study are included in the article. Further inquiries can be directed to the corresponding authors.

## Supplementary Materials

The following supporting information can be downloaded at: https://www.mdpi.com/article/10.3390/molecules31091446/s1, Figure S1: PCA Analysis of QC Samples in positive ion conditions; Table S1. Component identification results by UPLC-MS; Table S2: Component identification results by HS-GCMS; Table S3: Quality assessment parameters; Table S4: CSF biomarkers on the 30th day of DCI rats; Table S5: Metabolic pathway enrichment information on the 30th day of DCI rats; Table S6: CSF biomarkers on the 45th day of DCI rats; Table S7: Metabolic pathway enrichment information on the 45th day of DCI rats; Table S8: CSF biomarkers on the 60th day of DCI rats; Table S9: Metabolic pathway enrichment information on the 60th day of DCI rats; Table S10: CSF biomarkers on the 90th day of DCI rats; Table S11: Metabolic pathway enrichment information on the 90th day of DCI rats.

## Abbreviations

The following abbreviations are used in this manuscript:

DCI	diabetic cognitive impairment
SX	Shichangpu–Xiyangshen herb pair
CSF	cerebrospinal fluid
DM	diabetes mellitus
MWM	Morris water maze
AD	Alzheimer’s disease
MCI	mild cognitive impairment
mPGES	microsomal and perinuclear PGE synthase
5-LOX	5-Lipoxygenase
TCM	traditional Chinese medicine
STZ	streptozotocin
QC	quality control
PGE2	prostaglandin E2
AA	arachidonic acid
LTB4	leukotriene B4
TNF-α	tumor necrosis factor-alpha
IL-8	interleukin-8
qRT-PCR	quantitative reverse transcription–polymerase chain reaction
