# Supplementary material for: Shichangpu–Xiyangshen Herb Extract Alleviates Cognitive Dysfunction in Type 1 Diabetes Through Metabolism of Arachidonic Acid Cyclooxygenase and Lipoxygenase
Source: Molecules. 2026 Apr 27;31(9):1446. doi: 10.3390/molecules31091446 (PMC13165223; doi:10.3390/molecules31091446)
Supplement: Supplementary file 1 [file molecules-31-01446-s001.zip › molecules-4193128-supplementary.pdf]

Supplementary Material

Figure S1. PCA Analysis of QC Samples in positive ion conditions.

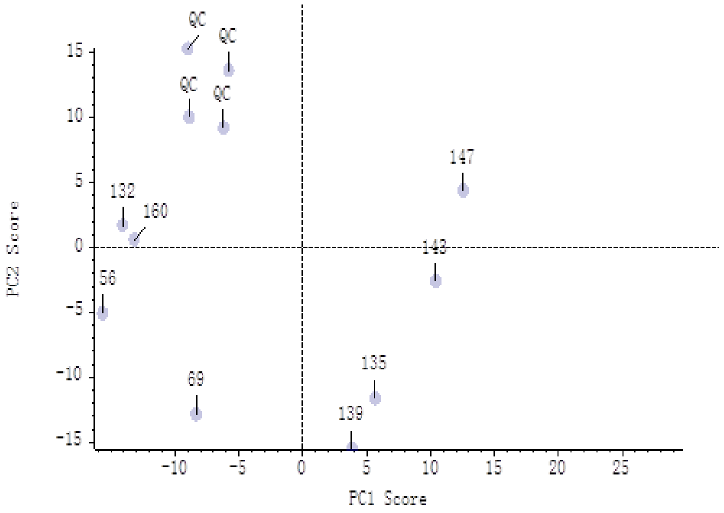

Table S1. Component identification results by UPLC-MS

No.	t_R/min	Formula	Observed（m/z）	Mass error (ppm)	MS/MS	Lon mode（ESI⁻/ESI⁺）	Component name
1	12.34	$C_{54}H_{92}O_{23}$	1107.6093	-1.80	945.5565; 783.4995;621.4455; 179.0563	ESI^-	Ginsenoside Rb1
2	12.63	$C_{53}H_{90}O_{22}$	1077.6008	-1.84	945.5523; 783.5008; 621.4580; 149.0441	ESI^-	Ginsenoside Rc
3	12.73	$C_{53}H_{90}O_{22}$	1077.6001	-1.85	945.5502; 915.5706; 783.5024; 621.4439; 293.0958; 251.0771; 149.0465	ESI^-	Ginsenoside Rb2
4	13.07	$C_{53}H_{90}O_{22}$	1077.5994	-1.85	945.5452; 783.5017; 621.4334	ESI^-	Ginsenoside Rb3
5	9.06	$C_{48}H_{82}O_{18}$	945.5532	-2.11	799.4942; 637.4363; 619.4260; 475.3826	ESI^-	Ginsenoside Re
6	9.24	$C_{48}H_{82}O_{18}$	945.5535	-2.11	799.4930; 783.4983; 637.4364; 619.4259; 475.3805; 161.0458	ESI^-	Ginsenoside Rd
7	11.97	$C_{42}H_{72}O_{14}$	799.4953	-3.15	653.4316; 635.4316; 635.4263; 491.3778	ESI^-	Ginsenoside Rg1
8	12.14	$C_{42}H_{72}O_{14}$	799.4942	-2.49	653.4323; 635.4241; 491.3766; 161.0461	ESI^-	Ginsenoside F11
9	11.62	$C_{42}H_{72}O_{14}$	801.4775	-0.02	783.4606; 439.3425 439.3425; 421.3318; 143.1041	ESI^+	Ginsenoside F11
9	17.02	$C_{42}H_{72}O_{13}$	783.4960	-2.55	621.4397; 459.3820; 161.0425	ESI^-	Ginsenoside Rg2

Table S2. Component identification results by HS-GCMS

No.	Formula	m/z	RT	Component name	Score
1	$C_{11}H_{14}O_2$	178.3	18.895	Methyleugenol	93.45
2	$C_{15}H_{24}$	161.2	20.674	δ-cadinene	86.78
3	$C_{12}H_{16}O_3$	208.4	21.081	γ-Asarone	91
4	$C_{15}H_{22}O$	105.1	22.804	(E)-Isovalencenal	85.8
5	C_7H_{14}	98.1	6.984	Cyclohexylmethane	84.5
6	$C_{10}H_{12}O$	148.1	16.053	Estragole	96.77
7	$C_{11}H_{14}O_2$	178.1	19.601	(Z)-Methylisoeugenol	96.44
8	$C_{12}H_{16}O_3$	208.1	21.615	β-Asarone	98.2

Table S3. Quality assessment parameters.

Samples	Ion model	Models	R2Xcum	R2Ycum	Q2cum
30 day	neg	PCA	0.760		
		OPLS-DA	0.346	0.969	0.931
	pos	PCA	0.510		
		OPLS-DA	0.301	0.961	0.897
45 day	neg	PCA	0.510		
		OPLS-DA	0.389	0.780	0.712
	pos	PCA	0.477		
		OPLS-DA	0.256	0.847	0.800
60 day	neg	PCA	0.556		
		OPLS-DA	0.379	0.901	0.821
	pos	PCA	0.307		
		OPLS-DA	0.157	0.622	0.415
90 day	neg	PCA	0.445		
		OPLS-DA	0.299	0.897	0.860
	pos	PCA	0.536		
		OPLS-DA	0.294	0.824	0.703

Table S4. CSF biomarkers on the 30th day of DCI rats.

NO.	Calculated m/z	Ionform	HMDB	Metabolite Name	Classify	Trend
1	295.2284	$[M+H]^+$	HMDB10220	9(10)-EpODE	lipide	↑
2	313.2387	$[M+H]^+$	HMDB10208	15,16-DiHODE	lipide	↑
3	782.5616	$[M+H]^+$	HMDB07889	PC(14:0/22:4(7Z,10Z,13Z,16Z))	lipide	↑
4	788.5172	$[M-H]^-$	HMDB09045	PE(18:1(11Z)/22:6(4Z,7Z,10Z,13Z,16Z,19Z))	lipide	↓
5	961.9211	$[M+H]^+$	HMDB43081	TG(15:0/20:0/24:0)	lipide	↓
6	149.0587	$[M+H]^+$	HMDB00567	Cinnamic acid	Phenylpropanoic acids	↑
7	209.0596	$[M+H]^+$	HMDB28784	Cysteinyl-Serine	organic acid	↑
8	215.104	$[M-H]^-$	HMDB29027	Prolyl-Threonine	organic acid	↓
9	191.0204	$[M-H]^-$	HMDB00193	Isocitric acid	organic acid	↓
10	537.1662	$[M+H]^+$	HMDB06944	1,4-beta-D-Glucan	organic oxide	↓
11	115.1114	$[M+H]^+$	HMDB04814	4-Heptanone	organic oxide	↓
12	206.0465	$[M-H]^-$	HMDB00978	4-(2-Aminophenyl)-2,4-dioxobutanoic acid	organic oxide	↓
13	206.0451	$[M+H]^+$	HMDB00881	Xanthurenic acid	organic heterocycle	↑
14	585.2736	$[M+H]^+$	HMDB00054	Bilirubin	organic heterocycle	↓
15	107.049	$[M+H]^+$	HMDB06115	Benzaldehyde	benzenoid hydrocarbons	↑
16	221.0824	$[M-H]^-$	HMDB13247	Monobutylphthalate	benzenoid hydrocarbons	↑
17	152.0361	$[M-H]^-$	HMDB01476	3-Hydroxyanthranilic acid	benzenoid hydrocarbons	↑
18	402.0857	$[M-H]^-$	HMDB06806	Propinol adenylate	nucleotide	↑
19	317.2122	$[M-H]^-$	HMDB0001337	Leukotriene A4	organic heterocyclic	↑
20	271.4260	$[M-H]^-$	HMDB0001220	Prostaglandin E2	organic heterocyclic	↑

* ↑ indicates that the increased of biomarkers in DCI group compared with the control group (p <0.05); ↓ indicates that the decreased of biomarkers in DCI group compared with the control group (p <0.05)

Table S5. Metabolic pathway enrichment information on the 30th day of DCI rats.

Pathway Name	Match Status	p	-log(p)	Holm p	FDR	Impact
Arachidonic acid metabolism	3/44	0.0014744	2.8314	0.11795	0.11795	0.09758
Glycerophospholipid metabolism	2/36	0.016203	1.7904	1	0.55415	0.19969
Tryptophan metabolism	2/41	0.020781	1.6823	1	0.55415	0.05157
Linoleic acid metabolism	1/5	0.027983	1.5531	1	0.55967	0
alpha-Linolenic acid metabolism	1/9	0.071311	1.1468	1	1	0
Citrate cycle (TCA cycle)	1/20	0.1078	0.96736	1	1	0.04498
Propanoate metabolism	1/22	0.11799	0.92814	1	1	0.04103
Porphyrin metabolism	1/31	0.16258	0.78893	1	1	0.0528
Glyoxylate and dicarboxylate metabolism	1/32	0.16741	0.77622	1	1	0
Glycosylphosphatidylinositol (GPI)-anchor biosynthesis	1/32	0.16741	0.77622	1	1	0.03665

Table S6. CSF biomarkers on the 45th day of DCI rats.

NO.	Calculated m/z	Ionform	HMDB	Metabolite Name	Classify	Trend
1	295.2275	[M+H]$^+$	HMDB10203	13-HOTE	Lipide	↑
2	313.2387	[M+H]$^+$	HMDB10208	15,16-DiHODE	Lipide	↑
3	295.2284	[M+H]$^+$	HMDB10220	9(10)-EpODE	Lipide	↑
4	295.2269	[M+H]$^+$	HMDB10206	15(16)-EpODE	Lipide	↑
5	219.1755	[M+H]$^+$	HMDB13687	Nookatone	Lipide	↓
6	221.1902	[M+H]$^+$	HMDB13688	Nootkatol	Lipide	↑
7	437.2658	[M-H]$^-$	HMDB07854	LPA(18:0/0:0)	Lipide	↓
8	1253.7727	[M+H]$^+$	HMDB04901	Ganglioside GA1 (d18:1/9Z-18:1)	Lipide	↓
9	165.091	[M+H]$^+$	HMDB01955	3-Phenylbutyric acid	Phenylpropionic acids	↑
10	102.0911	[M+H]$^{+-}$	HMDB12815	5-Aminopentanal	organic oxide	↓
11	184.9852	[M+H]$^+$	HMDB01024	Phosphohydroxypyruvic acid	organic oxide	↓
12	347.2237	[M-H]$^-$	HMDB12849	7'-Carboxy-alpha-tocotrienol	organic heterocycle	↑
13	585.2736	[M+H]$^+$	HMDB00054	Bilirubin	organic heterocycle	↓
14	206.0451	[M+H]$^+$	HMDB00881	Xanthurenic acid	organic heterocycle	↑
15	179.1182	[M+H]$^+$	HMDB01329	2'-Hydroxynicotine	organic heterocycle	↑
16	161.107	[M+H]$^+$	HMDB00303	Tryptamine	organic heterocycle	↑
17	305.1784	[M-H]$^-$	HMDB12799	5'-Carboxy-gamma-chromanol	organic heterocycle	↓
18	217.154	[M-H]$^-$	HMDB29140	Valyl-Valine	organic heterocycle	↓
19	263.1031	[M-H]$^-$	HMDB28974	Methionyl-Hydroxyproline	organic acid	↑
20	213.1239	[M+H]$^+$	HMDB11180	L-prolyl-L-proline	organic acid	↓
21	191.0204	[M-H]-	HMDB00193	Isocitric acid	organic acid	↓
22	143.105	[M+H]$^+$	HMDB31343	Methyl cyclohexanecarboxylat	organic acid	↓
23	107.049	[M+H]$^+$	HMDB06115	Benzaldehyde	benzenoid hydrocarbons	↑
24	152.0361	[M-H]$^-$	HMDB01476	3-Hydroxyanthranilic acid	benzenoid hydrocarbons	↑
25	317.2122	[M-H]$^-$	HMDB0001337	Leukotriene A4	organic heterocyclic	↑
26	271.4260	[M-H]$^-$	HMDB0001220	Prostaglandin E2	organic heterocyclic	↑

* ↑ indicates that the increased of biomarkers in DCI group compared with the control group (p <0.05); ↓ indicates that the decreased of biomarkers in DCI group compared with the control group (p <0.05)

Table S7. Metabolic pathway enrichment information on the 45th day of DCI rats.

Pathway Name	Match Status	p	-log(p)	Holm p	FDR	Impact
Tryptophan metabolism	2/41	0.020781	1.6823	1	0.9505	0.09148
Arachidonic acid metabolism	2/44	0.023762	1.6241	1	0.9505	0.09758
Glycerolipid metabolism	1/16	0.08711	1.0599	1	1	0.01246
Citrate cycle (TCA cycle)	1/20	0.1078	0.96736	1	1	0.04498
Porphyrin metabolism	1/31	0.16258	0.78893	1	1	0.0528
Sphingolipid metabolism	1/32	0.16741	0.77622	1	1	0
Glyoxylate and dicarboxylate metabolism	1/32	0.16741	0.77622	1	1	0
Cysteine and methionine metabolism	1/33	0.17221	0.76394	1	1	9.50E-04
Glycine, serine and threonine metabolism	1/33	0.17221	0.76394	1	1	0.02475
Glycerophospholipid metabolism	1/36	0.18648	0.72938	1	1	0.13895

Table S8. CSF biomarkers on the 60th day of DCI rats.

NO	calculated m/z	Ionform	HMDB	Metabolite Name	Classify	Trend
1	278.1505	$[M+H]^+$	HMDB28957	Lysyl-Methionine	organic acid	↑
2	174.1124	$[M+H]^+$	HMDB11756	N-Acetylleucine	organic acid	↓
3	302.1482	$[M+H]^+$	HMDB29028	Prolyl-Tryptophan	organic acid	↑
4	261.1454	$[M+H]^+$	HMDB11171	L-gamma-glutamyl-L-leucine	organic acid	↑
5	247.1283	$[M+H]^+$	HMDB28903	Isoleucyl-Aspartate	organic acid	↑
6	217.154	$[M+H]^+$	HMDB29140	Valyl-Valine	organic acid	↓
7	232.1298	$[M+H]^+$	HMDB29122	Valyl-Asparagine	organic acid	↓
8	283.1287	$[M+H]^+$	HMDB29073	Threoninyl-Tyrosine	organic acid	↓
9	231.1693	$[M+H]^+$	HMDB29130	Valyl-Isoleucine	organic acid	↓
10	762.6019	$[M+H]^+$	HMDB08262	PC(20:0/14:0)	Lipide	↓
11	766.5413	$[M+H]^+$	HMDB08944	PE(16:0/22:5(4Z,7Z,10Z,13Z,16Z))	Lipide	↓
12	784.5829	$[M+H]^+$	HMDB08041	PC(18:0/18:3(9Z,12Z,15Z))	Lipide	↓
13	734.5684	$[M+H]^+$	HMDB00564	PC(16:0/16:0)	Lipide	↓
14	806.5718	$[M+H]^{+-}$	HMDB08083	PC(18:1(11Z)/20:5(5Z,8Z,11Z,14Z,17Z))	Lipide	↓
15	664.5284	$[M+H]^+$	HMDB29206	lysoPC(28:0)	Lipide	↓
16	165.091	$[M+H]^+$	HMDB01955	3-Phenylbutyric acid	Phenylpropionic acids	↑
17	100.0764	$[M+H]^+$	HMDB11749	2-Piperidinone	organic heterocycle	↑
18	161.107	$[M+H]^+$	HMDB00303	Tryptamine	organic heterocycle	↑
19	305.1784	$[M-H]^-$	HMDB12799	5'-Carboxy-gamma-chromanol	organic heterocycle	↓
20	585.2736	$[M+H]^+$	HMDB00054	Bilirubin	organic heterocycle	↓
21	152.0361	$[M-H]^-$	HMDB01476	3-Hydroxyanthranilic acid	benzenoid hydrocarbons	↑
22	317.2122	$[M-H]^-$	HMDB0001337	Leukotriene A4	organic heterocyclic	↑
23	271.4260	$[M-H]^-$	HMDB0001220	Prostaglandin E2	organic heterocyclic	↑

* ↑ indicates that the increased of biomarkers in DCI group compared with the control group (p <0.05); ↓ indicates that the decreased of biomarkers in DCI group compared with the control group (p <0.05)

Table S9. Metabolic pathway enrichment information on the 60th day of DCI rats.

Pathway Name	Match Status	p	-log(p)	Holm p	FDR	Impact
Arachidonic acid metabolism	3/44	6.39E-04	3.1947	0.05109 9	0.051099	0.09758
Glycerophospholipid metabolism	2/36	0.0097248	2.0121	0.76826	0.33399	0.19969
Tryptophan metabolism	2/41	0.012525	1.9022	0.97692	0.33399	0.09148
Linoleic acid metabolism	1/5	0.02182	1.6612	1	0.43639	0
alpha-Linolenic acid metabolism	1/13	0.055882	1.2527	1	0.89412	0
Porphyrin metabolism	1/31	0.12883	0.89	1	1	0.0528
Glycosylphosphatidylinositol (GPI)-anchor biosynthesis	1/32	0.13273	0.87702	1	1	0.03665

Table S10. CSF biomarkers on the 90th day of DCI rats.

NO.	calculated m/z	Ionform	HMDB	Metabolite Name	Classify	Trend
1	939.4951	$[M+H]^+$	HMDB09932	PIP(16:0/20:4(8Z,11Z,14Z,17Z))	Lipid	↓
2	822.6458	$[M+H]^+$	HMDB08653	PC(22:4(7Z,10Z,13Z,16Z)/P-18:0)	Lipid	↑
3	734.5637	$[M+H]^+$	HMDB08899	PE(15:0/20:0)	Lipid	↑
4	766.5775	$[M+H]^+$	HMDB13415	PC(o-16:1(9Z)/20:4(8Z,11Z,14Z,17Z))	Lipid	↓
5	746.6057	$[M+H]^+$	HMDB13426	PC(o-18:1(9Z)/16:0)	Lipid	↑
6	810.5331	$[M+H]^+$	HMDB12404	PS(18:2(9Z,12Z)/20:3(8Z,11Z,14Z))	Lipid	↑
7	758.5969	$[M+H]^+$	HMDB11381	PE(P-18:0/20:1(11Z))	Lipid	↓
8	838.5332	$[M+H]^+$	HMDB09704	E(22:6(4Z,7Z,10Z,13Z,16Z,19Z)/22:5(7Z, 10Z,13Z,16Z,19Z))	Lipid	↑
9	854.6641	$[M+H]^+$	HMDB09568	PE(22:2(13Z,16Z)/22:1(13Z))	Lipid	↑
10	900.7485	$[M+H]^+$	HMDB08546	PC(22:0/22:1(13Z))	Lipid	↓
11	738.5046	[M-H]-	HMDB09061	PE(18:1(9Z)/18:3(6Z,9Z,12Z))	Lipid	↑
12	813.6838	$[M+H]^+$	HMDB12107	SM(d18:1/24:1(15Z))	Lipid	↑
13	165.091	$[M+H]^+$	HMDB01955	3-Phenylbutyric acid	Phenylpropioni c acids	↑
14	149.0597	$[M+H]^+$	HMDB00567	Cinnamic acid	Phenylpropioni c acids	↓
15	311.1229	$[M+H]^+$	HMDB11741	Gamma-Glutamyltyrosine	Organic acid	↑
16	217.154	$[M+H]^{+-}$	HMDB29140	Valyl-Valine	Organic acid	↓
17	283.1287	$[M+H]^+$	HMDB61146	Hydroxy-lacosamide	Organic acid	↑
18	189.1226	$[M+H]^+$	HMDB28700	Alanyl-Valine	Organic acid	↑
19	225.0352	$[M+H]^+$	HMDB28772	Cysteinyl-Cysteine	Organic acid	↑
20	591.3156	$[M+H]^+$	HMDB04158	D-Urobilinogen	Organic heterocycle	↓
21	307.1914	$[M+H]^+$	HMDB12799	5'-Carboxy-gamma-chromanol	Organic heterocycle	↓
22	161.107	$[M+H]^+$	HMDB00303	Tryptamine	Organic heterocycle	↑
23	338.0872	[M-H]-	HMDB10363	5-Hydroxy-6-methoxyindole glucuronide	Organic oxide	↓
24	384.1163	$[M+H]^+$	HMDB00912	Succinyladenosine	Nucleotide	↑
25	152.0361	[M-H]-	HMDB01476	3-Hydroxyanthranilic acid	Benzenoid hydrocarbons	↑
26	317.2122	$[M-H]^-$	HMDB0001337	Leukotriene A4	Organic heterocyclic	↑
27	271.4260	$[M-H]^-$	HMDB0001220	Prostaglandin E2	Organic heterocyclic	↑

* ↑ indicates that the increased of biomarkers in DCI group compared with the control group (p <0.05); ↓ indicates that the decreased of biomarkers in DCI group compared with the control group (p <0.05)

Table S11. Metabolic pathway enrichment information on the 90th day of DCI rats.

Pathway Name	Match Status	p	-log(p)	Holm p	FDR	Impact
Arachidonic acid metabolism	3/44	0.0014744	2.8314	0.11795	0.11795	0.09758
Glycerophospholipid metabolism	2/36	0.016203	1.7904	1	0.55415	0.19969
Tryptophan metabolism	2/41	0.020781	1.6823	1	0.55415	0.09148
Linoleic acid metabolism	1/5	0.027983	1.5531	1	0.55967	0
Ascorbate and aldarate metabolism	1/9	0.049867	1.3022	1	0.79786	0
alpha-Linolenic acid metabolism	1/13	0.071311	1.1468	1	0.95081	0
Pentose and glucuronate interconversions	1/19	0.10267	0.98855	1	1	0.10843
Porphyrin metabolism	1/31	0.16258	0.78893	1	1	0
Sphingolipid metabolism	1/32	0.16741	0.77622	1	1	0
Glycosylphosphatidylinositol (GPI)-anchor biosynthesis	1/32	0.16741	0.77622	1	1	0.03665
